# Effects of background doping, interdiffusion and layer thickness fluctuation on the transport characteristics of THz quantum cascade lasers

**DOI:** 10.1038/s41598-024-55700-7

**Published:** 2024-03-07

**Authors:** Novak Stanojević, Aleksandar Demić, Nikola Vuković, Paul Dean, Zoran Ikonić, Dragan Indjin, Jelena Radovanović

**Affiliations:** 1 School of Electrical Engineering, University of Belgrade, Belgrade, 11000 Serbia; 2 Vlatacom Institute of High Technologies, Belgrade, 11000 Serbia; 3 School of Electronic and Electrical Engineering, University of Leeds, Leeds, LS2 9JT UK

**Keywords:** Nanoscience and technology, Quantum cascade lasers

## Abstract

In this work, we investigate the effects of *n* and *p*-type background doping, interface composition diffusion (interdiffusion) of the barrier material and layer thickness variation during molecular beam epitaxy (MBE) growth on transport characteristics of terahertz-frequency quantum cascade lasers (THz QCLs). We analysed four exemplary structures: a bound-to-continuum design, hybrid design, LO-phonon design and a two-well high-temperature performance LO-phonon design. The exemplary bound-to-continuum design has shown to be the most sensitive to the background doping as it stops lasing for concentrations around $1.0\cdot 10^{15}$–$2.0\cdot 10^{15}$ cm^-3^. The LO-phonon design is the most sensitive to growth fluctuations during MBE and this is critical for novel LO-phonon structures optimised for high-temperature performance. We predict that interdiffusion mostly affects current density for designs with narrow barrier layers and higher $\textrm{Al}$ composition. We show that layer thickness variation leads to significant changes in material gain and current density, and in some cases to the growth of nonfunctional devices. These effects serve as a beacon of fundamental calibration steps required for successful realisation of THz QCLs.

## Introduction

The terahertz-frequency quantum cascade lasers (THz QCLs)^1,2^ emit light in the traditionally hard-to-reach far-infrared part of the spectrum. This has enabled increasing applications in medical diagnostics, chemical sensing and imaging^3–10^. The lasing frequency spans from 1.2 to 5.6 THz^11–14^, and the maximum achieved optical power at cryogenic temperatures larger than 1 W^15^. The maximum temperature at which pulsed operation was achieved was 261 K^16^, while continuous operation was achieved at 129 K^17^. Owing to the unique THz light emission of QCLs, many designs have been developed, the most important of which are the Longitudinal Optical Phonon (LO), Bound to Continuum (BTC), and hybrid designs^18^. Recently, novel design schemes have been proposed introducing a paradigm shift in designing LO-phonon structures with transitions farther from the resonant energy (36 meV in GaAs)^19,20^ and this has enabled high-temperature performance >250 K^16,21^.

The semi-classical rate-equation (RE) transport models^22^ were usually applied in the design of mid-infrared QCLs, but were unable to include coherent quantum mechanical transport effects, resulting in non-physical results in THz QCL designs^23,24^. On the other hand, the most general quantum models such as the Wigner function formalism^25^, or the non-equilibrium Green function (NEGF) model^26–28^ do take into account coherence effects, but tend to have a very high computational cost. In this work we use the density matrix (DM) model^24,29–32^, which is a versatile quantum transport model with low numerical complexity that includes coherence effects, and adequately describes resonant tunneling through the injection barrier, making it an ideal model for the intensive optimisation of THz QCL structures. The DM model that we employ^24,29^ uses the first neighbour and tight binding approximations, and employs the infinite period QCL consideration. Scattering mechanisms such as non-radiative electron interactions with longitudinal optical (LO) phonons, acoustic (AC) phonons, alloy disorder (AD), interface roughness (IFR), ionised impurities (II) and other electrons (EE) are described as perturbations with the Fermi-golden rule^33^.

THz QCLs are typically grown by molecular beam epitaxy (MBE) due to its high growth quality. THz QCLs consist of >100 periods of multiple-quantum-well heterostructures and are greatly affected by MBE process and its fluctuations. In this work, we study three effects associated with MBE growth that may have a prominent impact on THz QCL transport: background doping, interface composition diffusion (interdiffusion) and layer thickness fluctuations.

Background doping of unwanted *n* or *p*-type dopants in every layer of the structure is present due to impurities of the materials in the effusion cells or the impurities from the MBE machine itself. Even though background doping is an experimentally known effect^34^, its theoretical modeling is not present in literature to the best of our knowledge. In this work, we investigate the effect of both *n* and *p*-type background doping on transport characteristics in the three most common THz QCL designs in order to see how susceptible these designs are, and to determine the critical values of background doping at which electron transport is significantly affected.

QCL wafer growth by MBE is done at high temperatures, making them prone to interdiffusion of the added barrier material (Al in the case of $\mathrm {Al_{x}Ga_{1-x}As}$ structures). This results in realistic QCLs not having abrupt interfaces between layers, which can have a prominent effect on QCL operation^35^. In this work, we use the finite difference method for numerical solving of Fick’s law to model interdiffusion and investigate its effect on carrier transport.

The growth of QCLs by MBE is made layer by layer where the time of growth of each layer is calculated depending on the desired layer thickness and the incident molecule flux. Due to the variations of the flux intensity, the layers can be grown with different thicknesses than desired. In order to see the effect of layer thickness fluctuations on QCL transport we allowed random ±1 layer thickness fluctuations by one monolayer, for each layer. This was done to show how easily fluctuations of this nature during growth can change the desired QCL transport characteristics and as a demonstration of how the same QCL design grown multiple times can have different performance.

Finally, in our analysis, we have focused on how these effects affect the transport characteristics of the new high-temperature record QCL that has recently achieved pulsed operation at 261 K^16^. This is an important contribution because this design is novel in many aspects, its active region comprises only two quantum wells, the barriers have a higher Al mole composition than in conventional THz QCLs ($\mathrm {x=0.35}$) and the LO-phonon extraction energy is 48.5 meV which is significantly away from the 36 meV LO-phonon resonant energy and thus may exhibit greater fluctuations during MBE growth.

## Results

We present the results of our numerical simulations for three exemplary QCL designs: LO-phonon design, BTC design, and hybrid design^24,36,37^ as well as for the record operation temperature 261 K two-well LO-phonon QCL design^16^ which are separately analysed. The exemplary LO-phonon QCL achieved pulsed operation at 200 K^36^ with emission at 3.22 THz. The exemplary BTC QCL is designed for operation at a frequency of 2 THz^24^. The exemplary hybrid QCL is designed for low threshold power density and high power output emission at  3.9 THz operating in continuous-wave mode^37^, while the two-well high-temperature performance 4 THz LO-phonon design achieved operation at 261 K^16^. Detailed description of the active medium of all four QCLs can be found in the Sect. "Methods".

### Background doping

**Figure 1:**
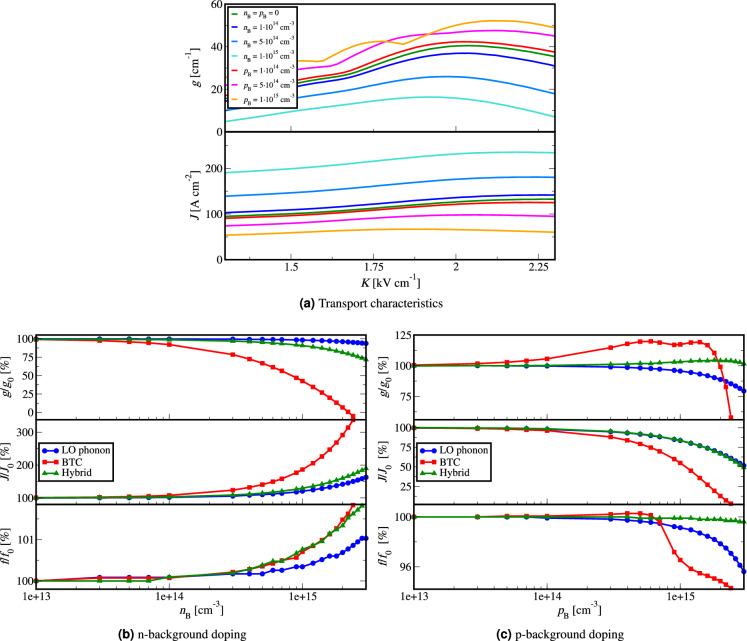
(**a**) Material gain and current density versus external bias for BTC QCL^24^ for different concentrations of *n* and *p* background doping. Material gain, current density and emission frequency for different QCL designs^24,36,37^ versus: (**b**) *n* background doping concentration, and (**c**) *p* background doping concentration presented relative to the values that each design had with no background doping at a chosen external bias around the maximum of the material gain (shown in Table 1).

We present the effect of background doping on the transport characteristics of exemplary THz QCLs by altering their doping profile as in Eqs. (1) and (2). Figure 1a shows how the material gain and current density versus applied external bias are affected by changing the background doping density in the case of the BTC QCL.

In the case of *n*-type background doping the total number of electrons is increased. This increases the current density leading to a decrease in the material gain relative to the case with no background doping in Fig. 1a. The *p*-type background doping conversely led to a decrease of the current density due to the recombination of the electrons and holes, which led to increase in the material gain.

We can conclude that *n*-type background doping would increase the current density and that *p*-type would decrease it. We cannot conclude generally how this would affect the material gain, because nominally, there is an optimal doping level for each THz QCL design. It is important to note that this means that background doping affects how much we would need to compensate for the nominal level of doping.

To determine the sensitivity of different types of THz QCL designs and background doping concentrations at which the transport characteristics are significantly altered we varied both the *n* and *p*-type background doping concentrations starting from 0 cm^-3^ up to $3.0\cdot 10^{15}$ cm^-3^ for three designs^24,36,37^. The results are presented in Fig. 1b and Fig. 1c and the presented values are relative to the values that each design had when no background doping is present at a chosen external bias around the maximum of the material gain. Values of material gain, current density, emission frequency and external bias for these three designs^24,36,37^ as well as the two-well high-temperature 261 K LO-phonon record design^16^ are shown in Table 1 for a specified external bias around the maximum of the material gain, as well as at the negative differential resistance (NDR) point.

**Table 1:** Material gain, current density and emission frequency for three exemplary QCL designs^24,36,37^ as well as for the record operation temperature 261 K LO-phonon QCL design^16^, at a specified external bias around the maximum of the material gain, and at the negative differential resistance (NDR) point.

QCL design	$g_0$ [cm^-1^]	$J_0$ [Acm^-2^]	$f_0$ [THz]	$K_0$ [kVcm^-1^]
LO-phonon 200 K	74.4	948.4	3.49	13.0
BTC	38.2	121.5	2.13	1.9
Hybrid	40.0	229.0	3.66	7.5
LO-phonon 261 K	64.2	2591.5	3.99	22.5
LO-phonon 200 K NDR	46.1	1291.2	3.77	14.3
BTC NDR	34.7	132.6	2.16	2.3
Hybrid NDR	34.3	234.5	3.73	7.8
LO-phonon 261 K NDR	65.0	2601.4	4.02	22.6

Figure 1b shows that in all three designs *n*-type background doping significantly increases the current density and slightly increases (up to $\sim$2%) the emission frequency. The material gain is significantly decreased for the BTC design making it the most sensitive design to *n*-type background doping, followed by the hybrid and lastly the LO-phonon design. The BTC design stops lasing around the background doping concentration of $1.0\cdot 10^{15}$ cm^-3^ where its material gain is reduced to the assumed threshold value of $20~\mathrm {cm^{-3}}$. The hybrid and LO-phonon designs have increased current, while their material gain is not so significantly reduced. This will result in more robust performance than the BTC design, at the cost of increased threshold current and electrical heating of the device in pulsed operation, and limiting or disabling continuous wave operation of hybrid structures.

In Fig. 1c we can observe that for all three designs *p*-type background doping significantly decreases (up to $\sim$5%) the current density and slightly decreases the emission frequency. For values around the background doping concentration of $2.0\cdot 10^{15}$ cm^-3^ the current density of the BTC design is reduced to around 0. The material gain of the BTC first increases as its current is slightly reduced, but is then significantly reduced as the current density drops. The LO-phonon design is more sensitive to *p*-type background doping than the hybrid design as its material gain decreased, whereas the hybrid design showed no significant change in material gain. In the case of *p*-type as well as *n*-type background doping, the BTC design stops lasing for the background doping concentration of around $1.0\cdot 10^{15}$ cm^-3^ to $2.0\cdot 10^{15}$ cm^-3^.

The physical explanation of what we observe in Fig. 1 can be inferred from the band structure of the devices we have analysed (shown in Fig. 5). The BTC design has a low threshold, low bias and typically operates at low frequency (<2.5 THz). This makes the BTC design very susceptible towards variations in the doping profile because the effects of the Hartree potential term are more significant than in LO-phonon and hybrid design (the bandstructure potential would bend more significantly with higher doping levels and this would directly affect the electron states and device performance). Additionally BTC designs have long periods, and since background doping is present in each layer, the magnitude of the sheet doping density would be higher than in designs with shorter periods. These two traits of BTC design make it more sensitive to background doping. In contrast, the LO-phonon design has a high threshold, high bias and a short period, thus the effect of Hartree potential is significantly lower than in the BTC design. However, the LO-phonon design requires more precise resonant state alignment (Fig. 5a) and since every THz QCL has an optimal doping level, the LO-phonon design shows moderate sensitivity to background doping. Finally, the hybrid design aims to combine the best traits of BTC and LO-phonon schemes: the depopulation of the lower lasing level is done through LO-phonon transition and fast diagonal mini-band relaxation, where the ground level may also be a mini-band to enable better injection of carriers into the upper lasing level in the next period. Therefore the hybrid designs operate at moderate bias, moderate threshold and have long periods^19^. Their sensitivity to background doping cannot be generally stated because they can lean more either towards LO-phonon designs or BTC designs. For example, a hybrid with a very long period would lean towards BTC design having a lower threshold^19^ and the Hartree potential contribution would be more significant. Converse arguments apply if a particular hybrid design leans more towards the LO-phonon design. A particular hybrid design may also be very robust to background doping (as in Fig.1) in the case when the Hartree potential contribution due to doping is weak (overcoming the shortcoming of BTC design) and pumping of the upper lasing level is more efficient than in LO-phonon design (overcoming the shortcoming of LO-phonon design).

We note that the model we presented in Eqs. (1) and (2) is phenomenological, however the background doping concentration values at which THz QCL transport is significantly impacted in Fig. 1b and c are consistent with experimental observations that estimate that background doping concentrations $>5\cdot 10^{14}~\mathrm {cm^{-3}}$ (*p*-type) are highly detrimental for THz QCL operation^34^.

### Interdiffusion

**Figure 2:**
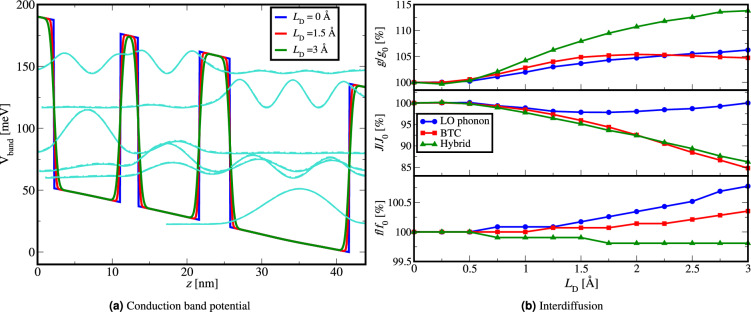
(**a**) Bottom of the conduction band potential of the 200 K LO-phonon THz QCL^36^ for different diffusion lengths of interdiffusion, (**b**) Material gain, current density and emission frequency versus diffusion length of interdiffusion for different QCL designs^24,36,37^ presented relative to the values that each design had with no interdiffusion, at a chosen external bias around the maximum of the material gain (Table 1).

The heterostructure interface cannot physically be abrupt because interdiffusion between well and barrier material affects the profile of the conduction band potential and its electronic structure. This can be numerically modelled through Fick’s law in Eq. (4) and this effect is typically characterised by diffusion length $L_D$. In this work, we will assume that all interfaces exhibit identical diffusion lengths. In Fig. 2a we illustrate how the conduction band profile of the exemplary LO-phonon structure is affected. By transferring the barrier material from the barrier layers to the well layers, interdiffusion effectively widens the top, and narrows the bottom of the well layers. This leads to a slight rise in the energy of the electron subbands which slightly alters the emission frequency depending on the diffusion length.

To examine the sensitivity of different THz QCL designs to interdiffusion, we vary the diffusion length from 0 Å up to 3.0 Å in the entire structure for all three exemplary designs^24,36,37^. In Fig. 2b we present results relative to the values that each design had with no interdiffusion, at a chosen external bias around the maximum of the material gain (Table 1).

In Fig. 2b we note that interdiffusion leads to an increase in the material gain and for the BTC and hybrid design a decrease in the current density. For all three designs, the emission frequency is negligibly affected. The decrease in the current density occurs due to the widening of the injection barrier which lowers the probability of resonant tunneling through the injection barrier into the upper laser level (ULL) of the next period, and thus lowers the tunnelling current.

### Layer thickness fluctuations

**Figure 3:**
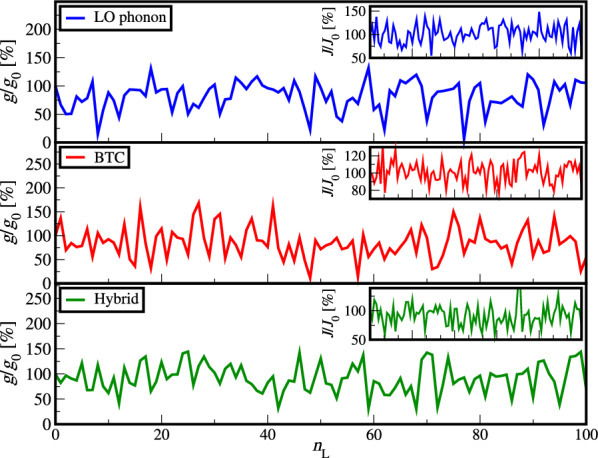
Material gain and current density (insets) for the first 100 random layer thickness fluctuations for all three QCL designs^24,36,37^ presented relative to non-perturbed values in Table 1.

The thickness of individual layers during MBE growth is determined by the time needed for their solidification, while in the transport model we typically use fixed spatial values. Additionally, the incident molecular flux can fluctuate during growth and desired thickness may not be achieved. From the modelling perspective, we typically only model one period of the device and this is justified for this effect, as it is a random process. We model this effect by introducing thickness deviation of ±1 monolayer (2.8 Å in $\mathrm {Al_{x}Ga_{1-x}As}$ system) in every layer of exemplary THz QCLs. In each variation every layer is either changed for ±1 monolayer or stayed the same. We simulated 500 random variations for which we calculated the mean values, standard deviation (STD) and root-mean-square error (RMSE) of the material gain, current density and emission frequency. We focus on bias values that correspond to the maxima of current density (NDR point) of each exemplary device and present our results relative to non-perturbed values in Table 1.  The material gain and current density for the first 100 random variations for all three exemplary QCL designs are presented in Fig. 3.

In Table 2 we present the mean value, standard deviation and RMSE of the 500 random variations for all three QCL designs.

**Table 2:** Mean value, standard deviation and RMSE of material gain, current density and emission frequency for three different QCL designs^24,36,37^ for 500 variations relative to the designed values at NDR point, as well as for the 261 K record high temperature LO-phonon QCL^16^ for all of the 81 possible layer thickness fluctuations relative to the designed values at NDR point.

QCL design	LO-phonon 200 K	BTC	Hybrid	LO-phonon 261 K
$g_{\textrm{mean}}/g_0$ [%]	84.0	86.8	95.3	92.1
$g_{\textrm{STD}}/g_0$ [%]	23.4	32.6	29.9	21.6
$(g_0 \pm g_{\textrm{RMSE}})/g_0$ [%]	$100 \pm 28.4$	$100 \pm 35.1$	$100 \pm 30.3$	$100 \pm 22.0$
$J_{\textrm{mean}}/J_0$ [%]	100.5	100.7	94.0	97.8
$J_{\textrm{STD}}/J_0$ [%]	20.1	13.1	20.7	27.5
$(J_0 \pm J_{\textrm{RMSE}})/J_0$ [%]	$100 \pm 20.1$	$100 \pm 13.1$	$100 \pm 21.6$	$100 \pm 27.7$
$f_{\textrm{mean}}/f_0$ [%]	93.1	99.8	97.8	106.0
$f_{\textrm{STD}}/f_0$ [%]	17.2	8.8	9.2	19.2
$(f_0 \pm f_{\textrm{RMSE}})/f_0$ [%]	$100 \pm 18.5$	$100 \pm 8.8$	$100 \pm 9.4$	$100 \pm 20.1$

We can observe that the material gain is the most affected as it has both the largest reduction in mean values, and the largest RMSE. It is followed by the current density, and lastly the emission frequency, which does not change significantly. Owing to this large variation of the material gain, a large number of the random variations of the structure have material gain bellow the threshold value and cannot lase. For a double-metal plasmonic waveguide the typical losses (for devices operating in frequency range 2 - 4 THz) are around 20 cm^-1^, which corresponds to relative values of material gain in Fig. 3 of 43.3 %, 57.6 % and 58.3 % for the LO-phonon, BTC, and hybrid design respectively.

Since we used random generated number sequence with values -1, 0 and +1 for every layer, each layer was statistically erroneous in 66.67 % cases. This means that the larger the number of layers the QCL design has, the greater is the number of faults that were made in the random variations. As the LO-phonon design has 6 layers, the BTC design 16 and the hybrid design 8 layers the average number of faults made in the random layer thickness fluctuations was 4, 10.67 and 5.33 respectively. While the results in Table 2 do show that the material gain RMSE of the LO-phonon design is slightly smaller than that of the BTC and hybrid design, it was achieved through a smaller average number of fluctuations, because of which the LO-phonon design shows to be the most sensitive to layer thickness fluctuations, followed by the hybrid design.

The reason for the greater sensitivity of the LO-phonon design and also, to a smaller extent of the hybrid design, is their dependence on the electron-LO-phonon scattering as the extraction mechanism.  The variation of the layer thickness changes the subband state energies which are originally designed to be spaced so that the QCL achieves operation through the electron-LO-phonon scattering mechanism. The BTC design achieves extraction from the lowest laser level (LLL) to the injection laser level (ILL) through diagonal transitions within the miniband of narrowly spaced states, which is more resistant to the shift of the subband energies, and can in turn withstand a greater number of faults and still operate. For the same reason, the hybrid design is more robust than the LO-phonon design as its LLL is actually a miniband that scatters towards ILL via electron-LO-phonon scattering mechanism.

We note that in Fig. 3 we may have illustrated effects of growth fluctuations of a poor quality MBE system. The typical tolerances of modern MBE systems used for THz QCL growth fluctuate well below $\pm 1$ monolayer thickness. The more likely, realistic effect is fluctuation of incident flux towards mid and late stages of QCL stack growth, which typically comprises >100 periods. This would mean that later periods in QCL stack may have different composition than the desired, and from a modelling perspective, this is challenging as it would lead to formation of electric field domains^38,39^ and non-uniform bias distribution, disabling periodic modelling approach. We can, however, conclude that growth fluctuations do limit growth and performance repeatability of QCL structures as the effect would be present even with MBE systems with low layer thickness tolerances.

### High temperature performance THz QCL structures

THz QCLs have operated below 200 K in pulsed operation^36^ until the new record was set initially to 210 K^40^ via high-barrier ($x=0.25$) two well LO-phonon design and more recently to 250 K and 261 K^16,21^ where higher barriers have been employed ($x=0.25-0.3$). The latter two records employ high LO-phonon depopulation energies (>48 meV) and in our previous works^19,20^, we have proposed similar novel designs where we argued that taller barriers and higher LO-phonon depopulation energies are beneficial for high temperature performance. Physically, electron-LO-phonon scattering is significant in range $10-70$ meV at high temperatures^20^ peaking at 36 meV in GaAs. The fundamental limit for high temperature performance is that non-radiative nature of LO phonon scattering would decrease population inversion between lasing states (in range $8-20$ meV). Throughout the history of development of QCL designs, LO-phonon scattering was ideal depopulation effect of LLL state and most designs which were optimised have resonant energy separation (36 meV) between LLL and ILL. This however is not the most efficient approach for high temperature performance application, because ULL may leak towards the ILL, and back-scattering effect (LO-phonon absorption between ILL and LLL) would also be more pronounced at LO-phonon resonant energy, thus reducing the efficiency of LLL depopulation. In Ref.^19^ we have proposed high temperature designs that employ $>45$ meV LLL - ILL energy separation, and also argued the benefits of tall barriers for high temperature performance.

The most recent experimental demonstrations of two well LO-phonon structures^16,21^ have demonstrated the success of the paradigm shift in THz QCL design. In this work, we want to separately analyze 261 K structure, because its barriers use $x=0.35$ Al content in the barriers, and energy separation between LLL and ILL is 48 meV (at resonant bias). We estimate that this design, and those similar to it, may be more susceptible to growth related effects we have discussed so far.

**Figure 4:**
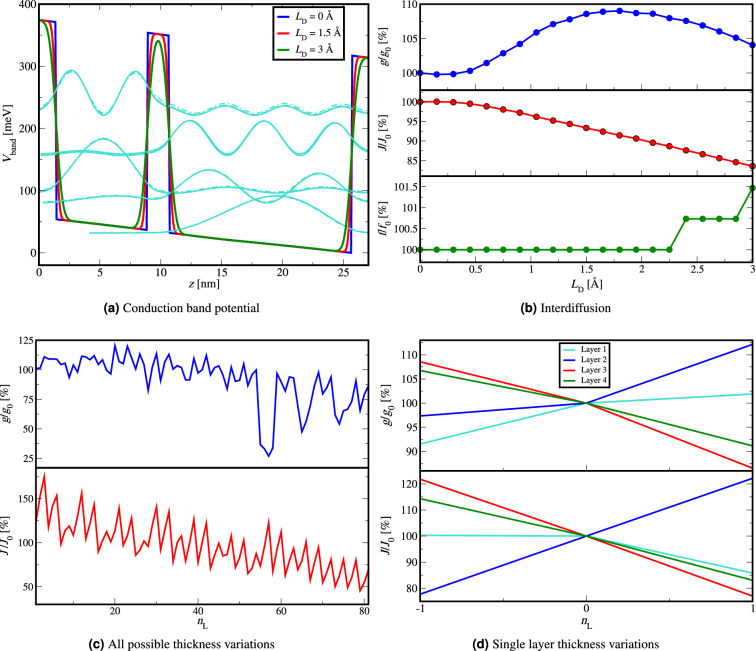
(**a**) Bottom of the conduction band of the 261 K record high temperature LO-phonon THz QCL^16^ for different diffusion lengths of interdiffusion, (**b**) material gain, current density and emission frequency versus diffusion length of interdiffusion for the 261 K record high temperature LO-phonon THz QCL^16^ shown relative to nominal device without interdiffusion around the maximum of the material gain (Table 1), (**c**) material gain and current density at NDR point for all of the 81 possible thickness variations for the 261 K record high temperature LO-phonon THz QCL^16^ presented relative to non-perturbed values in Table 1, (**d**) material gain and current density at NDR point for layer variations of only a single layer for all four layers in the 261 K record high temperature LO-phonon THz QCL^16^ presented relative to non-perturbed values in Table 1.

We first investigate the effect of interdiffusion on the 261 K record high-temperature LO-phonon THz QCL^16^. In Fig. 4a we illustrate how the conduction band profile of the structure is affected. Similarly as in Fig. 2b, in Fig. 4b we present the effect of interdiffusion on material gain, current density and emission frequency for this design, where the results are presented relative to the values that this design had for no interdiffusion at a chosen external bias around the maximum of the material gain. The values of material gain, current density, emission frequency and external bias for the nominally non-affected structure are shown in Table 1.

The results in Fig. 4b are consistent with observations in Fig. 2b but we can observe higher sensitivity than in exemplary 200 K LO-phonon structure we analysed previously. The most significant difference is that the 261 K structure experiences a large decrease in the current density when diffusion length is increased. This is attributed to significantly taller barriers of 261 K ($x=0.35$ and $\sim$317 meV in contrast to $x=0.15$ and $\sim$136 meV) and the fact that extraction energy separation between LLL and ILL is $\sim 48$ meV.  Owing to a larger Al content value and the larger barrier height, the subbands at higher energies are more affected by the interdiffusion, which can be seen in Fig. 4a. The resonant tunneling from the ILL into the ULL of the next period is more likely to be decreased by the widening of the injection barrier, lowering the tunnelling current, and this is more pronounced than in 200 K LO-phonon design in Fig. 2a.

The effect of background doping on the transport characteristics of the record high-temperature 261 K LO-phonon QCL is similar to the one shown previously for the exemplary QCLs in Fig. 1b and c, and as a result are not explicitly shown.

In Fig. 3 we have investigated the effect of potential growth fluctuations of $\pm 1$ monolayer thickness in each layer of the active region. Since the 261 K structure employs a two well design, there is a small number of layers in one QCL period, and as a result we are able to simulate all possible variations of the layer thicknesses and determine which layer is the most sensitive. The structure consists of a total of 4 layers and every layer variation has three possible values ($-1$, 0, +1) resulting in the total number of variations of $3^4=81$.

Figure  4c shows the relative material gain and current density for all of the 81 possible thickness variations for the 261 K record high-temperature LO-phonon QCL in the NDR point. The designed values of the structure without fluctuations in the NDR point are shown in Table 1, while the mean value, standard deviation and RMSE of material gain, current density and emission frequency are shown in Table 2.

We varied the thickness of each layer by $(-1,0,1) \cdot 2.825$ Å  by nesting four loops in our simulation code with possible counter values of -1, 0, and +1 where the order of the layers that corresponded to these counters from the outer to the inner loop was 3, 4, 1, 2, meaning that the outer loop controlled the value of the variation of the third layer, followed by the loop for the fourth layer, then the loop for the first layer (injection barrier) and then lastly the inner loop controlled the value of the variation of the second layer. Variation number 41 corresponds to the designed structure without layer thickness fluctuations as the counters for this variation are 0, 0, 0, 0. As a result of the outer loop controlling the value of the variation of the third layer, the first third of the variations (1-27) corresponds to layer variation value -1 of the third layer, the second third of the variations (28-54) corresponds to layer variation value 0 of the third layer and the last third of the variations (55-81) corresponds to layer variation value +1 of the third layer. Each of these three thirds has the same sequence of counters for the other three layers, differing only in the third layer. In this manner, we can directly observe the sensitivity to fluctuations of the third layer (which is the second barrier).

In Fig. 4c we can see that both material gain and current density decrease with the variation number, having the largest values in the first third of the variations when third layer was perturbed by -1 monolayer, and the lowest values in the last third of the variations when the third layer was perturbed by +1 monolayer. This shows us that for the 261K LO-phonon design the third layer is the most sensitive to layer thickness variation and its increase significantly lowers both the material gain and current density.

The physical reason for this is that the thickness of the second barrier controls how diagonal the lasing transition is, and affects the energy separation between LLL and ILL. By narrowing this layer, we slightly increase the dipole moment between lasing levels leading to higher material gain and current density, and conversely, by widening this barrier, dipole moment is reduced, leading to decrease of material gain current density.

We have also varied the thickness of a single layer with all the other layers having the fixed (originally designed) thickness in order to show the effect of each layer in the 261 K record high-temperature LO-phonon on the material gain and current density. The results in Fig. 4d are shown relative to the designed values without fluctuations shown in Table 1.

In Fig. 4d we can see that in terms of the material gain and current density the third layer is the most sensitive, followed by the fourth layer and the second layer. Layer thickness variation shifts the bound state energies changing the energy separation between the LLL and ILL, which affects the LLL extraction lowering the inversion population between the ULL and the LLL and in turn the material gain. The reduction of the current density is due to the increase of the energy spacing between the ILL and the ULL between which resonant tunneling occurs through the injection barrier.

## Discussion

We have analysed effects of *n* and *p* background doping, interdiffusion of the barrier material and layer thickness fluctuations on the transport characteristics of THz QCLs during MBE growth.

The *n*-type background doping increases the current density due to a larger number of electrons and decreases the material gain. The material gain is significantly decreased for the exemplary BTC design and stops lasing around the background doping concentration of $1.0\cdot 10^{15}$ cm^-3^, making it the most sensitive design to *n*-type background doping followed by the hybrid and lastly the LO-phonon design. The background doping of *p*-type reduces the current density owing to the recombination of electrons with holes, leading to an increase in the material gain, however structures may stop lasing if their current density is depleted. For *p* type background doping concentration of $2.0\cdot 10^{15}$ cm^-3^ the current density of the BTC design is reduced to around 0 making it the most sensitive to the effect.  The exemplary LO-phonon design displayed slightly higher sensitivity to this effect than the exemplary hybrid design.

The interdiffusion widens the bottom of the injection barrier thus decreasing the resonant tunneling effect, which lowers the injection current. The exemplary BTC and hybrid design showed to be more sensitive to interdiffusion than the exemplary 200 K LO-phonon design since some of their layers are very narrow and are relatively more affected by the same value of diffusion length.

The layer thickness fluctuations were examined by allowing up to one monolayer thickness fluctutation in each layer of the exemplary structures. We found that this can lead to a significant change in the material gain and current density of the QCLs, and in some cases to the growth of structures that cannot operate at all. Owing to the sensitivity of the LO-phonon scattering mechanism on the energy difference between energy subbands, designs that rely on this mechanism for extraction, such as the LO-phonon design followed by the hybrid design are more sensitive. The BTC design is more resistant to the shift of the subband energies, and can in turn withstand a greater number of fluctuations during the growth process and still operate. We note that tolerances of modern MBE systems are significantly lower than $\pm 1$ monolayer, and our simulations serve as a remainder of how sensitive THz QCL growth needs to be. We estimate that even at low fluctuation tolerances, this effect may affect growth repeatability of THz QCLs.

Finally, we have analysed the most recent 261K record high-temperature LO-phonon structure^16^ due to its unique electron structure and use of tall barriers. We observed significantly higher sensitivity to interdiffusion relative to exemplary LO-phonon structure^36^ that uses low barrier. We have also analysed the effect of layer thickness fluctuations in this design and found that the structure is highly sensitive to the thickness of the second barrier which controls how diagonal the lasing transition is.

The effects that we have discussed in this work are instrumental in realizing THz QCLs in other perspective material systems. The technology in $\mathrm {GaAs/Al_xGa_{1-x}As}$ material system is mature, however any other material system may suffer from high background doping levels or growth fluctuations. Similarly, the material systems that employ higher barriers would require thinner barrier layers which would cause detrimental interdiffusion. The analysis we conducted in this work aims to highlight these effects in $\mathrm {GaAs/Al_xGa_{1-x}As}$ material systems, and also serve as a beacon of fundamental calibration steps required for successful realisation of THz QCLs in other material systems.

## Methods

### Background doping

The background doping changes the doping profile and affects the Hartree term in the 1D effective mass Schrödinger equation, which is solved self-consistently with the Poisson equation in order to calculate the electronic structure in our model. Background doping also alters the total number of electrons in the structure, which affects the QCL transport as they are unipoloar devices.

In THz QCLs, typically the largest well is intentionally *n* doped with concentration $n_D$ and these carriers are responsible for device performance. To model the background doping, we will assume that all layers in the device are uniformly doped by additional dopants resulting in effective doping profile $n_{eff}$.

In the case of *n*-type background doping, the doping profile is altered by increasing the doping concentration in every layer for the background doping density $n_B$.

1 $$\begin{aligned} n_{eff}=n_D+n_B \end{aligned}$$

In the case of *p*-type background doping, the effect is more complex as the structure has both *n* and *p*-type dopants, and simply decreasing the doping concentration in every layer is not physically valid. The *p*-type dopants affect the valence band of the device, leading to increase of electron-hole recombination, which effectively reduces the electron concentration in the conduction band $n_D$. We employ a phenomenological model that alters the effective doping density $n_{eff}$ for the layers that are intentionally doped such that the overall carrier sheet density is equal to the carrier sheet density $N_D=n_D\cdot l_D$ reduced by the *p* background carrier sheet density $P_B=p_B\cdot l_{total}$.

2 $$\begin{aligned} n_{eff}=\frac{n_D\cdot l_D-p_B\cdot l_{total}}{l_D} \end{aligned}$$

where $l_D$ is the length of the intentionally doped layers and $l_{total}$ is the total length of one QCL active region period. The physical motivation for this is the fact that recombination of $n_D$ carriers with the *p*-type background dopants reduces the current density, which is taken into account through the reduction of the carrier sheet density. We note that at low temperatures, the background dopants may not be fully ionised, and our approach gives an estimate of the effect.

### Interdiffusion

The interdiffusion at heterostructure interface is modelled by numerically solving Fick’s law with the finite difference method for the $\textrm{Al}$ content composition *x*. In the case of 1D structures Fick’s law can be discretised as:

3 $$\begin{aligned} \frac{\partial x(z,t)}{\partial t}= & {} D_x\frac{\partial ^2 x(z,t)}{\partial z^2} \end{aligned}$$

4 $$\begin{aligned} \frac{x(z_i,t_{j+1})-x(z_i,t_j)}{dt}= & {} D_x\frac{x(z_{i+1},t_j)-2x(z_i,t_j)+x(z_{i-1},t_j)}{dz^2} \end{aligned}$$

where *dz* and *dt* are incremental values of the coordinate and time grid respectively. For each value of time, the composition is calculated for the entire structure using the values in the previous time value. This is repeated for a certain diffusion time *t* that is related to the diffusion length $L_D=\sqrt{Dt}$. By diffusing the composition, we have not only described a more realistic conduction band offset $U_c(z)$, but also the effective mass along the growth direction $m^{*}(z)$ which are both functions of the alloy composition *x*. The finite difference approach is in excellent agreement with the analytical error function results presented in Ref.^41^.

### Active medium description of the modelled QCLs

#### Exemplary 200 K LO-phonon THz QCL

The exemplary LO-phonon THz QCL achieved pulsed operation at a high temperature of 200 K^36^ with emission at 3.22 THz. The LLL is efficiently depopulated by longitudinal optical phonon scattering into the ILL, which is located at an LO phonon energy (36 meV in GaAs) below the LLL and that is strongly coupled to the ULL of the next period. Resonant tunneling occurs through the injection barrier depopulating the ILL. The layer thicknesses, starting with the injection barrier are **4.3**/8.9/**2.46**/8.15/**4.1**/(5.5+5.0+5.5) nm, where $\mathrm {Al_{0.15}Ga_{0.85}As}$ barriers are indicated in bold font, and the middle part of the last well doped to $6\cdot 10^{16}$ cm^-3^ is underlined.

**Figure 5:**
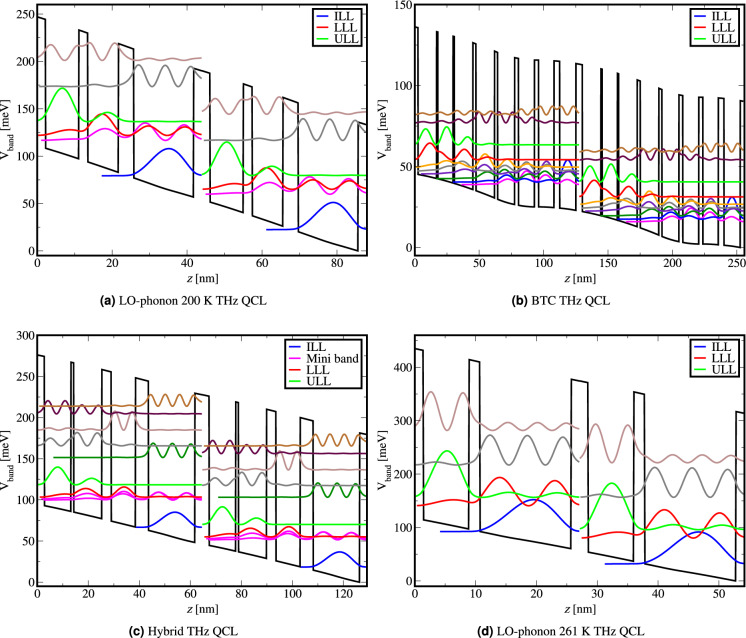
Bottom of the conduction band potential of the exemplary: (**a**) LO-phonon 200 K THz QCL^36^, (**b**) 2 THz BTC QCL^24^, (**c**) Hybrid THz QCL^37^, and (**d**) 261 K record high-temperature LO-phonon THz QCL^16^.

In Fig. 5a we show the bottom of the conduction band potential of the exemplary LO-phonon 200 K THz QCL^36^ with the wave functions squared shown.

#### Exemplary 2 THz BTC THz QCL

The exemplary BTC QCL is designed for operation at a frequency of 2 THz^24^. Extraction from the LLL to the ILL is achieved through diagonal transitions within the miniband, while the ILL is within the miniband and is strongly coupled to the ULL of the next period. The layer thicknesses, starting with the injection barrier are **5.0**/14.4/**1.0**/11.8/**1.0**/14.4/ **2.4**/14.4/**2.4**/13.2/**3.0**/12.4 /**3.2**/12.0/**4.4**/12.6 nm, where $\mathrm {Al_{0.1}Ga_{0.9}As}$ barriers are shown in bold, and wells doped to $1.3\cdot 10^{16}$ cm^-3^ are underlined.

In Fig. 5b we show the bottom of the conduction band potential of the exemplary BTC THz QCL^24^ with the wave functions squared shown.

#### Exemplary hybrid THz QCL

The exemplary hybrid QCL is designed for low threshold power density and high power output emission at  3.9 THz operating in continuous-wave mode^37^. The lasing occurs through a diagonal transition between the ULL and the LLL. The LLL is located above the miniband, which allows fast electron extraction. The miniband is depopulated to the ILL through LO-phonon scattering. The layer thicknesses, starting with the injection barrier are: **5.51**/10.39/**1.17**/10.92/**3.71**/9.54/**5.09**/(8.23+10.0) nm, where $\mathrm {Al_{0.2}Ga_{0.8}As}$ barriers are shown in bold, and the right part of the last well doped to $4.0\cdot 10^{16}$ cm^-3^ is underlined.

In Fig. 5c we show the bottom of the conduction band potential of the exemplary hybrid THz QCL^37^ with the wave functions squared shown.

#### Exemplary 261 K LO-phonon THz QCL

The 261 K record high-temperature LO-phonon THz QCL^16^ operating at 4 THz, employs $x=0.35$ Al content in the barriers and the LLL is efficiently depopulated by longitudinal optical phonon scattering into the ILL, which is located 48 meV below the LLL at resonant bias.

The layer thicknesses, starting with the injection barrier, are **2.88**/7.45/**1.76**/(3.0+3.0+9.0) nm, where $\mathrm {Al_{0.35}Ga_{0.65}As}$ barriers are indicated in bold font, and the middle part of the last well doped to $1.5\cdot 10^{17}$ cm^-3^ is underlined.

In Fig. 5d we show the bottom of the conduction band potential of the 261 K record high-temperature LO-phonon THz QCL^16^ with the wave functions squared shown.

### Calculating transport characteristics using density matrix model

We model the electron transport of THz QCL structures via DM model^24,29^ which generates transport parameters such as material gain *g*(*K*), current density *J*(*K*) and emission frequency *f*(*K*) as functions of the applied external electric bias *K*. Here we summarise the main steps of the algorithm used for calculating transport parameters.

First we set the lattice temperature and the electrical bias value, which correlates to terminal voltage of the device, and solve the Schrödinger-Poisson and kinetic balance equation (under equithermal subband approximation) self-consistently which results in the electron structure and electron temperature^42^.

Using the DM transport model we extract the material gain frequency dependence *g*(*f*) and current density value *J* . The peak of *g*(*f*) is set as the unsaturated material gain value and the corresponding frequency point as the lasing frequency. Using the same lattice temperature this process is repeated for a full range of applied electrical bias values.

The unsaturated material gain dependence on bias is extracted as *g*(*K*) and the current density dependence on bias is extracted as *J*(*K*). The peak of the *g*(*K*) corresponds to resonant tunnelling while the peak of *J*(*K*) corresponds to the negative differential resistance (NDR) point where experimentally the device typically stops lasing abruptly. The NDR point is a good indicator of the QCL characteristics as a large number of QCLs operate up to this point, and so it defines the maximum current density at which the QCL can operate, while operation starts for the current density value at which the material gain is equal to the threshold value. The corresponding bias value of the NDR point is extracted as $K_{\textrm{NDR}}$.

We illustrate the procedure by applying the algorithm on the 2 THz BTC laser^24^.

**Figure 6:**
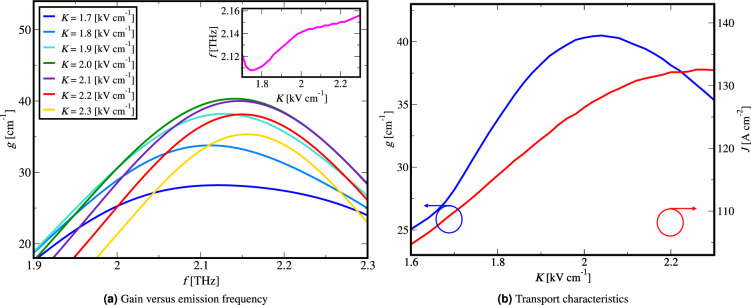
(**a**) Material gain dependence on emission frequency for different electric bias. The inset in the figure shows the emission frequency of the peaks of material gain versus the electric bias, (**b**) material gain and current density dependence on electric bias.

In Fig. 6a the material gain dependence on emission frequency *g*(*f*) is shown for different electric bias values. The peak of *g*(*f*) corresponds to the lasing mode for the applied electrical bias, and by extracting these peaks for different bias values the material gain dependence on electrical bias *g*(*K*) is found. By extracting the current density value for each electrical bias we find *J*(*K*). The inset in Fig. 6a shows the emission frequency of the peaks of material gain versus the electric bias *f*(*K*). The shift of the traces in Fig. 6a to higher frequencies as the bias increases is due to the Stark effect.

In Fig. 6b we present the calculated material gain *g*(*K*) and current density *J*(*K*) dependence on electrical bias.

## Data Availability

The data associated with this paper are openly available from the University of Leeds Data Repository. (https://doi.org/10.5518/1489).
